# Acceptability of an online theory‐based intervention to support healthcare professionals' delivery of health behaviour change interventions: A theoretically framed qualitative study

**DOI:** 10.1111/bjhp.70087

**Published:** 2026-07-06

**Authors:** Chris Keyworth, Katharina Sophie Vogt, Mark Conner, Christopher J. Armitage, Tracy Epton, Judith Johnson

**Affiliations:** ^1^ School of Psychology University of Leeds Leeds UK; ^2^ Department of Psychology, Institute of Population Health University of Liverpool Liverpool UK; ^3^ Manchester Centre for Health Psychology, School of Health Sciences University of Manchester Manchester UK; ^4^ Manchester University NHS Foundation Trust, Manchester Academic Health Science Centre Manchester UK; ^5^ NIHR Greater Manchester Patient Safety Translational Research Centre University of Manchester Manchester UK; ^6^ Division of Nursing, Midwifery and Social Work, Faculty of Biology, Medicine and Health, School of Health Sciences University of Manchester Manchester UK

**Keywords:** healthcare professionals, implementation intentions, qualitative, theoretical framework of acceptability

## Abstract

**Background:**

Healthcare professionals in the UK's National Health Service (NHS) are encouraged to deliver health behaviour change interventions during routine consultations. ‘If‐then’ planning helps healthcare professionals to work with patients to identify and overcome barriers. However, in order to deploy such interventions, healthcare professionals must find them acceptable.

**Aims:**

To use the Theoretical Framework of Acceptability (TFA) to understand the acceptability of interventions to support delivery of if‐then planning interventions by: (a) exploring healthcare professionals' experiences of an online theory‐based intervention which was designed to support them to deliver opportunistic health behaviour change interventions during routine clinical consultations, and (b) understanding the most prominent aspects of intervention acceptability to make recommendations for refinements and implementation.

**Method:**

Twenty‐six NHS healthcare professionals (including nurses, physiotherapists, dieticians, doctors and midwives) participated in semi‐structured interviews. The TFA informed both the design of the interview topic guide and the framework analysis, in which findings were mapped onto the TFA domains.

**Results:**

Five TFA domains were prominent: affective attitude, burden, intervention coherence, perceived effectiveness, and ethicality. There were mixed attitudes, but HCPs were broadly positive (affective attitude), valued the brief format (burden), and felt it aligned with their professional values (ethicality). They understood the intervention (intervention coherence) and believed it addressed barriers to health behaviour change conversations (perceived effectiveness).

**Conclusions:**

The intervention was broadly acceptable to healthcare professionals, with key domains including affective attitude, burden, intervention coherence, perceived effectiveness and ethicality identified as important. These findings provide specific targets for refining the intervention and supporting its implementation in routine healthcare practice.

**Statement of Contribution**

What is already known on this subject?

- Healthcare professionals face barriers to delivering behaviour change advice.
- If‐then interventions can support behaviour change across health contexts.
- Little is known about the acceptability of interventions for healthcare professionals.

What does this study add?

- If‐then planning was broadly acceptable to healthcare professionals.
- Five TFA domains explained intervention acceptability.
- Brief, flexible tools were valued in routine healthcare practice.

## INTRODUCTION

Noncommunicable diseases account for 41 million deaths every year, which equates to around 74% of deaths worldwide (World Health Organization, 2023). Noncommunicable diseases include (chronic) respiratory disease, heart disease, liver disease, diabetes, stroke, and cancer. Behavioural risk factors, such as smoking, lack of exercise, poor diet, and alcohol consumption, have been found to drastically increase an individual's likelihood of developing such noncommunicable diseases (Katzmarzyk, 2022; World Health Organization, 2023). The prevention of disease is thus, alongside the treatment of disease, now an integral part of healthcare professionals' roles (Forward, 2017). This is reflected in public health policies, which compel healthcare professionals to deliver opportunistic health behaviour change advice during routine consultations, encouraging alcohol reduction, smoking cessation, increased physical activity, and better diet in routine consultations with patients (Meade et al., 2023; Whitlock & Whitlock, 2002).

In the UK, the ‘Making Every Contact Count (MECC)’ policy is a key example of this approach, aiming to embed behaviour change interventions within the pre‐existing infrastructure of routine patient‐professional healthcare contact (Public Health England, 2016). Policy makers anticipated such policies would lead to substantial reductions in the incidence of noncommunicable diseases without the need for additional investment (Ion, 2011). There is evidence that opportunistic behaviour change interventions delivered during routine consultations can lead to improvements in a range of health behaviours by patients, including smoking cessation, weight management, and physical activity (Aveyard et al., 2016; Keyworth et al., 2020a; Webb et al., 2016).

However, both awareness of the MECC policy and its implementation into practice have been insufficient (Nelson et al., 2013). The reasons for this are complex and varied, including a lack of awareness of the policy, doubts about the effectiveness of opportunistic behaviour change interventions, a concern that offering advice outside an area of expertise is inappropriate, a lack of time to deliver opportunistic health behaviour change advice, increased workloads, and worries about jeopardizing patient–healthcare professional relationships (Haighton et al., 2021; Harrison et al., 2022; Pallin, 2022; Parchment et al., 2021).

The COVID‐19 public health emergency has influenced the delivery of opportunistic behaviour change interventions. A qualitative study with 25 healthcare professionals working in the NHS in the UK identified that while some barriers to delivering opportunistic health behaviour change interventions remained the same (lack of awareness, doubts about efficacy and ethicality, lack of time, workloads, worries about jeopardizing relationships), there were additional barriers, such as exacerbated staffing issues and poor platforms or IT equipment, which may limit the quality and continuity of patient interactions and reduce opportunities to deliver brief opportunistic health behaviour change interventions (Vogt et al., 2023). At the same time, the pandemic created new opportunities for the delivery of behaviour change interventions within routine care. In that study, healthcare professionals reported that using remote platforms to deliver healthcare services (including opportunistic behaviour change interventions) enabled more honest, less awkward conversations about health behaviour change (Vogt et al., 2023). Considering that remote consultations continue to be part of healthcare delivery, alongside traditional face‐to‐face consultations, this is a promising finding. However, many barriers persist, and healthcare professionals require support in overcoming these barriers to ensure they can deliver health behaviour change advice in line with the MECC policy.

### The intervention

The current study sought to assess the acceptability of a brief online intervention designed to support healthcare professionals in delivering MECC‐recommended opportunistic health behaviour change interventions during routine consultations, regardless of whether these are conducted face‐to‐face or remotely. The intervention is based on implementation intentions, a self‐regulatory strategy derived from theoretical models of goal striving, often operationalized as ‘if‐then’ plans to support behaviour change. If‐then plans work by making automatic links in memory between a critical situation (‘If I think a patient would benefit from a weight management intervention…’) and an appropriate response (‘…then I will signpost to a local support service’) (Gollwitzer, 1993, 1999). The rationale is that these if‐then statements become automatic responses when individuals are faced with the situation. Implementation intentions are an effective strategy for supporting behaviour change by helping individuals translate intentions into action (Gollwitzer, 1999; Gollwitzer & Sheeran, 2006). Traditionally, when making these plans, individuals can generate their own solutions to the problems/situations. However, this is often challenging to complete as people may be unable to form solutions themselves. A volitional helpsheet provides a list of situations that individuals may encounter and offers a range of appropriate responses or solutions to the situation. The barriers and corresponding solutions included in the volitional help sheet were informed by previous qualitative research examining barriers and facilitators to healthcare professionals' delivery of behaviour change interventions in routine practice (Vogt et al., 2023). Nine barrier–solution pairs were selected to provide coverage of key challenges while maintaining usability and brevity of the tool in routine practice. In this study, the volitional help sheet was delivered via a website, allowing participants to interact with and complete the tool online. Volitional help sheets have been found to be effective in a number of studies, including alcohol consumption, smoking, physical activity and self‐harm (Armitage & Arden, 2010, 2012; O'Connor et al., 2017), but have not been tested in the context of supporting healthcare professionals to deliver behaviour change interventions.

Intervention acceptability is recognized as a key consideration in intervention development (Sekhon et al., 2017; Skivington, 2021), and both implementation and subsequent intervention effectiveness may be dependent on perceptions of acceptability (Diepeveen et al., 2013; Sekhon et al., 2017). Research in a number of contexts such as support for public health policy and treatment adherence (Hommel et al., 2013; Stok, 2015) suggests that interventions perceived as acceptable are more likely to result in favourable outcomes.

The Theoretical Framework of Acceptability (TFA) was developed to guide the assessment of the acceptability of interventions, with acceptability being regarded as a multifaceted construct (Sekhon et al., 2017). The TFA consists of seven domains: affective attitude (how participants feel about taking part in the intervention), burden (how much effort is required to engage with the intervention), perceived effectiveness (whether individuals perceive an intervention as likely to achieve its goals), ethicality (the extent to which an intervention fits with a person's values), intervention coherence (whether individuals understand the intervention), opportunity costs (what needs to be given up to take part in the intervention), and self‐efficacy (confidence in doing the intervention). In comparison to more general approaches to assessing acceptability, the TFA allows a comprehensive and rich assessment of intervention acceptability, and allows intervention developers and researchers to understand opportunities to make iterations to interventions based on the specific TFA domains (Sekhon et al., 2017).

### Aims of the current study

The TFA has been used to guide data collection and analysis in qualitative studies across a range of contexts including health behaviour and long‐term health conditions (Liptrott et al., 2020; Pavlova et al., 2020). However, there is limited research applying the TFA to understand the acceptability of interventions that support healthcare professionals to deliver health behaviour change during routine consultations. Consequently, we aimed to apply the TFA to: (a) explore people's experiences of an online theory‐based if‐then intervention for healthcare professionals, which was designed to support them to deliver opportunistic health behaviour change interventions during routine clinical consultations, and (b) understand the most prominent aspects of intervention acceptability, to make recommendations for refinements and implementation of the intervention.

## METHODS

### Design and participants

A qualitative study design, with semi‐structured telephone interviews, was used with a range of healthcare professionals working in the NHS in the UK. Participants had previously taken part in a larger study (Keyworth et al., 2025) examining the effectiveness of a brief behaviour change intervention to support healthcare professionals in delivering behaviour change interventions during routine healthcare. The main trial provided evidence that the intervention was associated with improvements in healthcare professionals' delivery of health behaviour change interventions over time. The intervention was delivered via a website, which served as the platform for accessing an interactive version of the volitional help sheet (the core intervention component). In the larger study, participants were asked to complete the volitional help sheet via the website, generating ‘if‐then’ plans to support behaviour change conversations; this was intended as a general planning tool rather than something to be completed for each individual patient. The Theoretical Framework of Acceptability (TFA) was used to structure the assessment of the intervention's acceptability (Sekhon et al., 2017). Healthcare professionals with a patient‐facing role working in the NHS in the UK were eligible to take part in the study. Participants were recruited by YouGov on behalf of the research team from those who had taken part in the larger study and were allocated to the intervention group. A total of 26 healthcare professionals agreed to take part in the interviews. The sample was heterogeneous, comprising a range of healthcare professionals including nurses, doctors, physiotherapists, dietitians, and midwives, with variation in roles, experience, and clinical settings (Table 1) (DiCicco‐Bloom & Crabtree, 2006). Interviews were conducted following participants' use of the intervention as part of the larger study; participants had typically engaged with the website at least once prior to interview. Interviews were conducted approximately one year after participants had initially accessed the intervention, and participants were shown the intervention again during the interview to support discussion and reflection on their experiences of using it.

**TABLE 1:** Sample characteristics.

Name	Healthcare profession	Sex	Age
Ad	Mental Health Nurse	Male	45–50
An	Healthcare Assistant (Mental Health Wards)	Female	35–40
Ca	Diabetes Specialist Nurse	Female	55–60
Cl	Mental Health Nurse	Female	60–65
Cr	Psychologist in Early Intervention Psychosis	Male	35–40
Eb	Staff Nurse (Gynaecological Services)	Female	45–50
Ct	Occupational Therapist	Female	60–65
Er	Pharmacist in GP	Female	35–40
Ge	Pharmacist	Female	35–40
Jn	Staff Nurse	Female	25–30
Jo	GP	Female	35–40
Lo	GP Trainee	Female	25–30
Em	Mental Health Nurse	Female	50–55
Pl	Physiotherapist	Male	35–40
Pv	GP	Male	45–50
Ra	Specialist Nurse in CAMHS ADHD	Female	45–50
Ro	Community Learning Disability Nurse	Female	55–60
Rt	Nurse Practitioner in Sexual health	Female	50–55
Ru	Physiotherapist	Female	40–45
Sam	Mental Health Nurse	Female	50–55
Sh	Advanced Clinical Practitioner	Female	40–45
Sr	GP	Female	40–45
Tm	Nurse Specialist Cardiology	Male	55–60
Yu	Pharmacist	Male	25–30
Tg	Consultant in Geriatrics	Male	35–40
Sy	Paediatric Emergency Consultant	Female	50–55

### Data collection

Ethical approval was obtained from a University Ethics Committee. Data collection was conducted by YouGov, a survey panel company who regularly conduct research with healthcare professionals, in order to ensure that a purposively sampled range of healthcare professionals from a range of disciplines could be sampled.

Interviews were conducted by members of the YouGov qualitative methods team who were sufficiently trained in conducting qualitative interviews with healthcare professionals. Interviewers were provided with an interview topic guide (Appendix S1) and summary information sheet about the study for reference. Interviewers were encouraged to (a) use open‐ended questions, (b) be cautious when asking about current practice to mitigate social desirability or professional identity biases, and (c) ask participants to provide examples of experiences using the intervention in routine practice (where applicable). Invitation emails were distributed by YouGov, who incentivized potential participants in accordance with their points‐based reward system, whereby respondents accumulate points for taking part in online surveys, which can be exchanged for cash or voucher‐based rewards in line with standard panel practices (YouGov, 2018). The interviews were recorded and transcribed verbatim by the company, then anonymized and securely delivered to the research team for analysis. YouGov conducted the interviews and transcription only; all data analysis was conducted independently by the research team. Informed consent was obtained before each interview. No personally identifiable participant data was shared with the research team to adhere to YouGov's GDPR regulations. Prior to the interview, as part of an online questionnaire, participants were re‐presented with a web‐based version of the volitional help sheet, containing nine situations representing barriers to delivering behaviour change interventions, and nine possible solutions (ClinicalTrials.gov Identifier: NCT05820282). Participants were free to make as many situation–solution links as they desired. Figure S1 presents the volitional help sheet used in the intervention, including the situations and response options available to participants.

### Analysis

A directed content analysis approach (Hsieh & Shannon, 2005), which is suitable when using an existing theoretical framework to interpret the data, was used to identify and categorize instances of the TFA domains (Murphy & Gardner, 2019). Principles of the framework approach (Gale et al., 2013) were used to inform data analysis, with the seven components of the theoretical framework of acceptability as the framework (Sekhon et al., 2017). Deductive coding was used to organize the data in line with each of the TFA domains (Hsieh & Shannon, 2005). This involved reading each transcript and coding occurrences relating to each TFA domain. Analysis involved coding each occurrence in the interviews of each of the seven TFA domains, using the definitions accompanying each domain (Sekhon et al., 2017). This was repeated for each of the TFA domains. Domains were considered prominent where they were discussed frequently across interviews and supported by sufficiently rich data to enable meaningful interpretation.

## RESULTS

A total of 26 healthcare professionals were recruited as participants to the study by YouGov. Seven participants were male, 19 were female. Participant characteristics and age groups are represented in Table 1. Five prominent TFA domains (namely, affective attitude, burden, intervention coherence, perceived effectiveness, and ethicality) were represented in the analysis. Although all seven TFA domains were applied during coding, only five were sufficiently represented in the data to support detailed analysis. Opportunity costs and self‐efficacy were only briefly mentioned or not discussed at all. This approach is consistent with previous applications of the TFA, where only domains with rich qualitative content were reported (e.g., McIlroy et al., 2022; Rantala et al., 2023; Toomey et al., 2021).

### Affective attitude

Participants had mixed attitudes towards the intervention. Those who liked it praised it as *‘straightforward’*, ‘intuitive’, and user friendly, providing helpful solutions and alternatives to barriers and problems they may encounter when discussing health behaviour change with patients. Participants also said that the if‐then plans gave a structure of how to approach having conversations about health behaviour change.

*‘It just gives you the answer to your question really. It's really easy to use, it's simple to understand. It gives you the answers basically.’* – Ca; Diabetes Specialist Nurse.

*‘It was useful as a reminder of the different options that there were for you, because I think sometimes you can go in a cycle of going, 'Well, I'll just do this, I'll just refer to another health professional all the time,' like, when you're at work. So, I think you do forget about websites and the more external things, so it was a good reminder for that.’* – Jn; Staff Nurse

*‘If there's a problem, it seems to have a solution to it. Somebody's obviously been chatting with people in real life and coming up with what you need to do, because all the solutions are things you have done in the past, but to have them all written down at the same time, and be able to look at them and read through it*.’ – Ge; Pharmacist.

Others did not see the value in using the intervention as they were already having conversations about health behaviour change with their patients, and confident in their communication abilities.

*‘it's a form created for the sake of a form. It's a hoop created for the sake of a hoop and if you're a competent healthcare professional, who knows how to do things, and knows how to communicate with the patient, you don't need any of this.’* – RT; Nurse Practitioner in Sexual health.

Participants commonly reported that the website served as a reminder to have conversations about health behaviour change more broadly, as they realized, they were not doing enough of these, rather than to develop if‐then plans.

*‘It keeps that awareness at the forefront of your mind. It makes you more likely to be thinking in that frame of mind with each patient intervention, and it makes sure you keep up to date with other people that you can refer to.’* – Eb; Staff Nurse (Gynaecological Services).

*‘I think the one good thing about it is it's going to bring people's attention to even thinking about the person's overall lifestyle, like, general health, rather than just focusing on, like, 'Oh, they've come with a sore throat today.'‘* – Sh; Advanced Clinical Practitioner.

A further number of participants also said that they had their own strategies of how and when to discuss health behaviour change with patients, and that their own intuitive methods of communicating about this strongly resembled if‐then plans. Some said that because the intervention resembled what they were doing in practice so closely, they have not considered the intervention since.

*‘I'd probably do an if‐then plan, but not call it an if‐then plan. I've been doing things like that naturally for years*.‘– Rt; Nurse Practitioner in Sexual health.

Across interviews, participants reflected on whether some (and which) healthcare professions might be better placed to deliver opportunistic health behaviour interventions. While some said that they can see the intervention being useful to all healthcare professionals, others said that the intervention is only useful in settings where there is more time allocated for patient‐staff interactions, where the focus is on ‘holistic’ care. In these settings, it was suggested that healthcare professionals and patients can establish good relationships which then provide a context for delivering behaviour change advice.

‘I think it would be useful to me and my organisation and the department that I work in, you know, the emergency department side of things.’ – Sy; Paediatric Emergency Consultant.

In addition, some older, more experienced healthcare professionals felt that the intervention did not hold any value for them but would potentially be more suitable for less experience colleagues who may need reminders of how to approach health behaviour change conversations and overcome potential barriers.

*‘I think it's useful for newly qualified, and those starting off in healthcare.’* – Er; Pharmacist in GP.

*‘I think it would be a good tool as well for more newly qualified staff who maybe don't have as much knowledge of all the different options as well.’* – Jn; Staff Nurse.

*‘For GP trainees just about to qualify this would definitely a useful tool for them.’* – Pv; GP.

### Burden

A lack of time to discuss health behaviour change was reported as common. Most participants said that the website was quick to use and ‘*not too burdensome’* . As participants made general if‐then statements that could apply to conversations with different patients (rather than completing if‐then plans for each individual patient encounter), the intervention was not seen as time‐consuming. Participants described using the intervention to develop responses that could be applied across multiple patient interactions, rather than needing to complete the tool repeatedly. However, participants discussed that as with most tools in healthcare, practice would be important to embed if‐then plans into their daily routine.

‘*if you had to do this for every single patient that you saw, then yes, obviously it would take a significant amount of time. But if you've already internalised those messages and you know that you know where to signpost them for a healthy lifestyle conversations, or you know how to source the alternative languages if there's a language barrier or whatever, then you don't need to keep opening this every time, you just know it already.’* – SY; Paediatric Emergency Consultant.

*‘The process was easy, there was no hassle or there was no problem with it at all. I was able to look for the tool, and it helped me quickly to, you know, come up with a solution, so I thought it was a good tool.’‐* An; Healthcare Assistant (Mental Health Wards).

Thus, participants described the primary burden not as using the intervention itself, but as finding time to engage in conversations about health behaviour change within routine practice. One participant pointed out that collating the information and resources they would send to patients, as part of their if‐then plan focused around signposting, would take time in the first instance, but it would save time in the long‐run as the healthcare professionals would then have the resources ready for when they need them.

*‘Obviously, you would have to put the legwork in to finding what to signpost to in your local area. […] It's nice to have something formal to work with, rather than coming up with all entirely your own ideas. Yes, as I say, it saves a lot of time.’* – Ge; Pharmacist.

### Ethicality

Overall, participants discussed that offering behaviour change advice was in line with their value system, whether they used the if‐then plans or not. However, discussion around health behaviour change was limited by perceptions of external factors such as patient characteristics and constraints of the healthcare systems and the teams the participants were operating in. While participants who did not view the intervention positively did not typically comment on ethicality, those who liked it appeared to value it in part because it aligned well with their professional values and sense of responsibility.

*‘I don't think it would do any harm to anybody who are using this.’* – Pl; Physiotherapist

*‘Because we believe in trying to make things better for people, and that's what the plan is about, more or less.’* – Em; Mental Health Nurse.

*‘I think it completely fits with the type of professional that I am really. I mean, we're a regulated profession, one of the literal first standards that we're held accountable to is making patients your first priority, and I feel we should always be trying to strive to be the best that we can to deliver the best outcomes for our patients really. So, if there are ways that I can help about doing that then I'm all for it really.’* – Yu; Pharmacist.

### Intervention coherence

Generally, participants reported an understanding of how the intervention worked. Some wanted further clarification whether it was to be used with each patient, or as a tool that can be drawn on more generally. Most participants felt the website was easy and quick to access, and easy to understand. However, participants made suggestions for improving the delivery of the intervention, particularly in relation to the functionality, content, and integration of the website through which it was accessed. Participants also wanted the website to store links to other relevant websites, so they could have a database of specific websites to signpost patients to, brochures to give out, or services to refer to.

*‘It would just make life just a bit easier for me if I don't need to, kind of, find these things out [like other websites to signpost people to] myself*’ – Lo; GP Trainee.

*‘Could they provide an option for allowing people to free type if they have another way? There might be something that someone has thought of that's not written there.’* – Er; Pharmacist in GP.

Suggestions, such as integrating the website into the ‘systems’ at work, such as making it available as a Desktop shortcut on computers or turning the volitional help sheet into a phone app for easy access, were made, to ensure it is being accessed.

*‘I would probably like to keep it on my desktop, like, something I can shortcut, that I can just quickly go to. […] I think it would be good inbuilt within the system. The system we already have, if it was inbuilt more likely refer then for some people than having to go into your emails and find this email. I tend to use more tools that are already on the system, yes.’* – Er; Pharmacist in GP.

Some participants said that they wanted a way to record the if‐then plans in patients' notes for the following reasons: as a reminder for themselves to make every contact count, to follow the advice up with the patient at a later time themselves, to evidence they have given opportunistic behaviour change advice and to share their if‐then plans with other healthcare professionals involved in the patient's care. However, these suggestions may reflect a misunderstanding of the intended use of if‐then planning. Rather than being a tool to repeatedly return to, if‐then plans are typically designed to support the formation of automatic responses to specific cues—that is, to build habits. From the interviews, it was not always clear whether participants meant only recording those specific barriers they experienced with a particular patient, or sharing their personal, completed if‐then plans with colleagues via notes.

*‘Put it into the patient's record would be really good because another GP could then follow up with it at a later date* [*…*] *with patients and say, I can see what's being recorded here. GPs said they want to discuss it at the next visit, so, yes. I mean, that's useful too, having that that you could put in the notes that also helps as an audit tool as to the effectiveness of it.’* – Sr; GP.

*‘… just record it and then at least then I will know for next time, 'Well, I did this. Did it work?' If need be, I can go back to it again and, 'Oh, I tried that, it didn't work. Okay, what were the other things I should've or could've tried?'*‘– Tg; Consultant in Geriatrics.

Participants also commented how important trust and manager endorsement is when encouraging healthcare professionals to use interventions, and that, following this approval, advertisement and training for the intervention would be needed that goes beyond sending an email to staff, as this is perceived as inefficient by participants, ideally with a focus on clarifying the purpose of if‐then planning as a habit‐forming strategy.

### Perceived effectiveness

Participants had mixed views around perceptions on the efficacy of the intervention. While some were positive that it would help them initiate health behaviour change conversations and overcome barriers, others felt that constraints, such as workloads and a lack of time, remained the same and reduced the likelihood of them offering behaviour change interventions. Participants also pointed out that not all options would apply to everyone, and that some solutions would not work in their settings or the population they work with, or they would be uncomfortable with some of the solutions provided.

*‘Signposting a patient to usable websites, like, my patients are predominantly, like, 80, 90 and aren't very technologically literate* [*…*] *and then, the healthy lifestyle service in the community… would be amazing, but we don't have one.’* – Sh; Advanced Clinical Practitioner.

*‘In learning disabilities* [*…*] *it might not be quite as relevant* [*…*] *as it would be for somebody who's average functioning.’* – Cr; Psychologist in Early Intervention Psychosis.

*‘it kind of depends on where they work, because some areas it just wouldn't be appropriate. I feel like it's obvious but places like ITU* [*…*], *they probably wouldn't really use this*.’ – Jn; Staff Nurse.

Furthermore, some participants raised issues about the intervention being too simplified, and the options offered on the website being too simplistic and not reflecting the complexities encountered in clinical practice.

*‘A lot of patients we encounter nowadays will be quite complex. They'll have lots of social needs and other stuff going on that can't be, I guess, fit into one solution only if that makes sense. So, I think that's where the complexity of, you know, real patients come in and then trying to… simplify in quotations marks against one solution.’* – Yu; Pharmacist.

*‘A lot of my patients, they engage in unhealthier lifestyle behaviours for very psychological reasons. So, okay, say someone is depressed, and they're over‐eating because they're depressed. If you don't tackle that depression, then you can do any of this stuff that you like, it's not going to get you anywhere. And that's a big thing in a lot of the patients that I've come across. And it's not captured in something like this.’* – Sh; Advanced Clinical Practitioner.

## DISCUSSION

This study aimed to explore the acceptability of a theory‐based intervention to support healthcare professionals in delivering opportunistic health behaviour change interventions. The TFA was applied to understand the most prominent aspects of acceptability, and to make recommendations for both future refinements of the intervention and how best to incorporate the intervention into routine practice for healthcare professionals. This is the first time the TFA has been applied to understand the acceptability of an intervention to support healthcare professionals in delivering health behaviour change interventions. There are two key contributions. First, this study provides insight into the acceptability of the intervention by identifying the five prominent TFA domains (namely, affective attitude, burden, intervention coherence, perceived effectiveness, and ethicality) from qualitative analysis of healthcare professionals' experiences. These domains provide further insight into the acceptability of the intervention and may help to further increase the effectiveness of the intervention. Second, the study offers practical recommendations for how the intervention could be incorporated into routine healthcare practice.

There were mixed attitudes towards the intervention (affective attitude). Some participants reported the intervention was straightforward and easy to use, which is consistent with the views of participants using the intervention in other health contexts (Hwang et al., 2023; Keyworth et al., 2022). The brief nature of the intervention reported by participants in the present study goes some way to addressing barriers around time that are often reported in the research (Haighton et al., 2021; Meade et al., 2023; Parchment et al., 2021). However, some participants believed the value of the intervention may be restricted to those healthcare professionals who were not already having conversations with patients about health behaviours. One possible explanation may be that our intervention is not applicable to all healthcare professionals, but rather just those who are currently not having health behaviour change conversations with patients. This is in line with research suggesting the important role that reflective and automatic processes (Potthoff et al., 2022; Presseau et al., 2014) may have in healthcare professional practice. This may suggest a more targeted approach to selecting participants who are not already delivering behaviour change interventions for training in the intervention could be beneficial.

The brief nature of the intervention was cited as a key factor in using the intervention (burden). Healthcare professionals reported that the intervention was not too time‐consuming and could be incorporated into routine consultations. This is in line with previous research showing brief interventions that can be incorporated into routine healthcare appear to be effective (Aveyard et al., 2016). More specifically, the present findings suggest if‐then plans could be used for patients who share similar characteristics or behaviours, or for common clinical situations or barriers encountered in practice, rather than constructing separate if‐then plans for each individual patient. This reflects the use of the intervention as a general planning tool to develop responses that can be applied across different patient groups and contexts, rather than being limited to specific conditions. Future research could therefore examine whether particular if‐then plans would be more suited to certain patient characteristics (for example, older versus younger patients, physically active versus physically inactive patients).

Participants valued behaviour change advice as part of routine practice and saw the intervention as a good fit with these values (ethicality). However, some noted that systemic constraints within the NHS, such as time pressures and competing priorities, could undermine the intervention's perceived value and its use in practice. Whilst the role of individual and organizational values in implementation is well documented (Parchment et al., 2021), our findings emphasize the persistent tension healthcare professionals experience between delivering ideal care and navigating real‐world system limitations.

Healthcare professionals generally understood the intervention and how it worked (intervention coherence). However, while they were able to navigate and use the tool, their understanding of the underlying behavioural mechanism—specifically how if‐then plans are intended to support habit formation—appeared limited. Similarly to perceived burden, some healthcare professionals wanted clearer instructions around how the intervention could be used in practice, such as with each patient or a broader training tool that could be drawn upon more generally. Healthcare professionals also described additional features and functionality that would be helpful, such as storing additional resources and information such as links to helpful websites to signpost patients to, or patient information leaflets to provide to patients. Incorporating the intervention into existing IT systems and patient notes was also seen as important. Technology‐based approaches to patient care, including a more joined‐up IT systems approach, have been identified as important features of successful implementation of interventions into healthcare settings (Keyworth et al., 2018). Future research could explore the practicalities of this approach further, including addressing the barriers and enablers to using technology‐based interventions for healthcare professionals (Borges do Nascimento et al., 2023). Although participants may not have fully grasped the intended use of if‐then planning as a strategy to automate responses to specific cues, this may not necessarily be a limitation. It is possible that using the tool repeatedly in practice could still lead to the desired habit formation over time.

Mixed views were reported with respect to perceived efficacy of the intervention (perceived effectiveness). Some healthcare professionals suggested the intervention would help overcome a number of barriers to engaging in health behaviour change conversations. However, others felt the effectiveness and implementation success may be hindered by constraints associated with working within the healthcare system. Intervention development and implementation must be considered in light of healthcare professional workload and complexities of the healthcare system such as organizational constraints (Parchment et al., 2021) which are often cited in previous research.

### Implications for practice

Our findings suggest that the ‘if‐then’ planning intervention is broadly acceptable to healthcare professionals in supporting health behaviour change conversations with patients. There are four specific recommendations which may increase the likelihood of successful implementation in routine practice. First, the intervention may be particularly beneficial for healthcare professionals not currently discussing health behaviours with patients as part of routine practice, thus suggesting a more targeted approach to selecting participants for training, for example through self‐report measures or audit of routine practice to identify healthcare professionals who may benefit most from additional support. Second, the intervention may be used to develop generalizable if–then plans for common patient types or clinical situations, allowing healthcare professionals to apply pre‐planned responses across multiple consultations rather than generating new plans for each individual patient. Third, organizations may need to actively support and prioritize health behaviour change discussions as part of routine care, including promoting the use of brief theory‐based interventions within clinical practice. Fourth, future work should explore ways of embedding the intervention into existing IT systems, such as patient notes and records.

Research suggests the impact of the COVID‐19 pandemic may be long lasting with respect to healthcare delivery (Greenhalgh et al., 2020; Lifford et al., 2024; Murphy et al., 2021), including how health behaviour change is part of routine medical consultations, which declined during the pandemic compared to previous levels (Keyworth et al., 2024). Research suggests more focus should be placed on understanding how health behaviour change can be incorporated into time restricted medical consultations, and particularly how this can be integrated into current practice and routine (Keyworth et al., 2020b). It is encouraging that healthcare professionals in the present study found an implementation‐intention based intervention broadly acceptable, and one that could be feasibly incorporated into time‐restricted consultations with patients. Technology‐based solutions may be particularly promising for supporting healthcare professional practice (Keyworth et al., 2018), and understanding how these can become more effectively embedded within practice is a crucial area for future research (McMillan et al., 2018). With further refinements, such as additional functionality to encourage signposting to resources and support services, and providing additional materials to be used within the consultation, this may help to further improve the usability and implementation of the intervention in routine practice.

### Strengths and limitations

The TFA (Sekhon et al., 2017) provides a robust theoretical basis to inform two important areas: (1) the development of future iterations of the volitional help sheet to support healthcare professionals deliver health behaviour change interventions during routine practice, and (2) we provide specific recommendations for increasing the successful implementation of the intervention into healthcare practice. Five specific domains emerged as important factors associated with intervention acceptability (affective attitude, burden, intervention coherence, perceived effectiveness, and ethicality), and using the TFA has allowed a detailed assessment of how each of these domains impact upon acceptability compared to more general approaches to assessing acceptability. The sampling frame enabled a range of views from diverse healthcare professional groups working across specialisms with varying roles and responsibilities, enhancing the depth and richness of the data (DiCicco‐Bloom & Crabtree, 2006). Thus, our theoretical framework allowed a synthesis of views across all professions on the barriers and enablers to delivering behaviour change interventions in light of pandemic‐related stressors.

The study has limitations. Healthcare professionals in the study had previously taken part in a cross‐sectional survey examining healthcare professional delivery of behaviour change interventions, and were thus a self‐selecting sample. The sample were also part of a pre‐existing sample of healthcare professionals who were recruited and incentivized through a survey panel company (YouGov). Consequently, the sample may not be fully representative of all healthcare professionals as there may be additional views not captured in the present study. The sample was predominantly female, which may be considered a limitation. However, this broadly reflects the gender distribution of the NHS healthcare workforce, particularly in patient‐facing roles. Whilst the TFA provides a robust theoretical basis for future research and practice, it is important to acknowledge that there are other analytical approaches that focus more explicitly on implementation (e.g., frameworks such as Normalization Process Theory, which examines how new interventions and ways of working become routinely embedded in everyday practice (May et al., 2009)) that may generate additional insights beyond those captured in the present study.

## CONCLUSION

In conclusion, a brief intervention based on implementation intentions is broadly acceptable in supporting healthcare professionals to deliver health behaviour change interventions in routine practice. Our findings suggest perceptions of acceptability are explained by five TFA domains: affective attitude, burden, intervention coherence, perceived effectiveness, and ethicality. Further refinements could still be made to the intervention, but despite this, it provides a useful tool for healthcare professionals to construct their own personalized implementation intentions to incorporate into routine healthcare delivery.

## AUTHOR CONTRIBUTIONS

**Chris Keyworth:** Conceptualization; formal analysis; funding acquisition; writing – original draft; writing – review and editing. **Katharina Sophie Vogt:** Conceptualization; formal analysis; writing – original draft; writing – review and editing. **Mark Conner:** Conceptualization; funding acquisition; writing – review and editing. **Christopher J. Armitage:** Conceptualization; funding acquisition; writing – review and editing. **Tracy Epton:** Conceptualization; writing – review and editing. **Judith Johnson:** Conceptualization; formal analysis; funding acquisition; writing – original draft; writing – review and editing.

## Supporting information


Appendix S1.

**Figure S1.** The volitional help sheet.

## Data Availability

The data that support the findings of this study are available on request from the corresponding author. The data are not publicly available due to privacy or ethical restrictions.
