# Supplementary material for: Acceptability of an online theory‐based intervention to support healthcare professionals' delivery of health behaviour change interventions: A theoretically framed qualitative study
Source: Br J Health Psychol. 2026 Jul 6;31(3):e70087. doi: 10.1111/bjhp.70087 (PMC13338650; doi:10.1111/bjhp.70087)
Supplement: Supplementary file 1 — Appendix S1. [file BJHP-31-0-s002.docx]

**Interview topic guide developed by [The University of Leeds] for YouGov January 2023**

**Background info for YouGov:**

**What is the purpose of this study?**
***Overview of the research***

Research suggests that COVID-19 has impacted the likelihood of applying the Making Every Contact Count (MECC) principles in healthcare professionals’ day-to-day routine practice as a healthcare professional. MECC, a National Health Service initiative, emphasises the prevention of health problems being at the heart of every NHS contact. The framework aims to use the millions of day-to-day interactions that healthcare professionals have with patients to offer concise healthy lifestyle information, encourage people to change their behaviour and to direct them to local services that can support them. Examples are:

- Directing people, where appropriate, to weight management clinics
- Offering healthy living advice such as dietary recommendations
- Supporting patients, where appropriate, to achieve weight loss

***The purpose of our study is to explore your experiences of taking part in a new intervention aiming to encourage you to think of ways of talking to patients about health behaviours (e.g. smoking cessation, improving diet, doing more physical activity). The intervention you took part in as part of a recent YouGov survey involved asking you to create if-then” plans. These work by making automatic links in memory between a particular situation (“If I think a patient would benefit from a weight management intervention...”) and an appropriate response (“...then I will signpost to a local support service”).***

**DEFINITION OF HEALTH BEHAVIOUR CHANGE: talking about improving diet, doing more physical activity, quitting smoking, losing weight, or reducing alcohol intake**

**Intro text – standard YG text (3 mins)**

- Hello and thank you for taking part in this interview today, which is being conducted by YouGov on behalf of [The University of Leeds].
- Remind them that the interview will be made anonymous and we follow the GDPR. Explain that their responses would only be reported via their job role and not their name
- Interview will be audio recorded for note-taking purposes. Are you happy for us to share the recording with the research team
- There are no right or wrong answers, please try to be as open and honest as possible. You may refuse to answer any question at any time.

**Please start by introducing yourself – 1^st^ name and role / main responsibilities.**

- **How long have you been in your current role?**
- **How long have you been working as an NHS healthcare professional in total?**

| **Attitudes towards making IF-THEN plans** |
| --- |
| - How did you feel about taking part in the intervention?   - What were the positive aspects of making IF-THEN plans to encourage you to think of ways of talking to patients about health behaviours?   - Were there any challenges with making IF-THEN plans?   - Is action planning something you find helpful? Have you tried action planning previously? |
| **Burden of making IF-THEN plans** |
| - How much effort was required to make your IF-THEN plans?   - How were they incorporated into your routine consultations with patients, if at all?     - Could you provide an example of how you did this? |
| **Ethicality of making IF-THEN plans** |
| - How much was forming IF-THEN plans a good fit with your personal values? How do you feel about making IF-THEN plans generally?   - Probe good fit versus challenging to align them with personal values |
| **Intervention coherence** |
| - To what extent do you understand IF-THEN plans and how they work?   - What aspects were clear? Were there any parts that were unclear? Could you suggest any improvements to the intervention? |
| **Opportunity costs** |
| - To what extent did you give up any benefits, profits or values to make your IF-THEN plans, and to engage in these plans? |
| **Perceived effectiveness** |
| - How confident were you / are you that your IF-THEN plans are likely to support you and other healthcare professionals in encouraging you to think of ways of talking to patients about health behaviours? |
| **Self-efficacy** |
| - How confident were you about making your IF-THEN plans to support you in thinking of ways of talking to patients about health behaviours? - How confident are you about maintaining your IF-THEN plans? |

*topic guide to be modified iteratively to probe emerging issues
