# Supplementary figures and images for: Acceptability of an online theory‐based intervention to support healthcare professionals' delivery of health behaviour change interventions: A theoretically framed qualitative study
Source: Br J Health Psychol. 2026 Jul 6;31(3):e70087. doi: 10.1111/bjhp.70087 (PMC13338650; doi:10.1111/bjhp.70087)

**Supplementary Figure 1.** The volitional help sheet


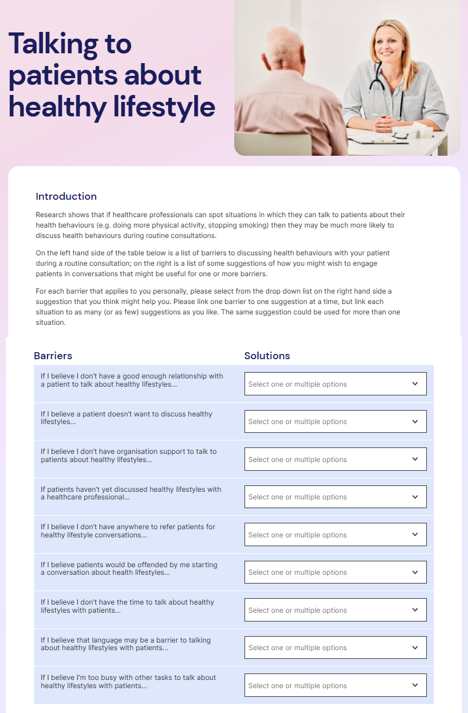

Supplement: Supplementary file 2 — Figure S1. The volitional help sheet. [file BJHP-31-0-s001.docx]
